# Estimating haplotype frequencies in pooled DNA samples when there is genotyping error

**DOI:** 10.1186/1471-2156-6-25

**Published:** 2005-05-19

**Authors:** Shannon RE Quade, Robert C Elston, Katrina AB Goddard

**Affiliations:** 1 Department of Epidemiology and Biostatistics, Case Western Reserve University, 2103 Cornell Rd, Cleveland, Ohio 44106-7281, USA

## Abstract

**Background:**

Maximum likelihood estimates of haplotype frequencies can be obtained from pooled DNA using the expectation maximization (EM) algorithm. Through simulation, we investigate the effect of genotyping error on the accuracy of haplotype frequency estimates obtained using this algorithm. We explore model parameters including allele frequency, inter-marker linkage disequilibrium (LD), genotyping error rate, and pool size.

**Results:**

Pool sizes of 2, 5, and 10 individuals achieved comparable levels of accuracy in the estimation procedure. Common marker allele frequencies and no inter-marker LD result in less accurate estimates. This pattern is observed regardless of the amount of genotyping error simulated.

**Conclusion:**

Genotyping error slightly decreases the accuracy of haplotype frequency estimates. However, the EM algorithm performs well even in the presence of genotyping error. Overall, pools of 2, 5, and 10 individuals yield similar accuracy of the haplotype frequency estimates, while reducing costs due to genotyping.

## Background

Association studies offer several advantages to linkage analysis for mapping susceptibility loci in complex diseases. They may be more powerful than linkage analysis for loci with a small effect, since the excess sharing across families is expected to be greater than the excess sharing within a family (identity-by-descent (IBD)) [1]. In addition, association studies are expected to provide greater precision in pinpointing the location of susceptibility loci. Finally, association studies do not require the collection of groups of relatives or extended pedigrees, which can be challenging – particularly for late onset diseases.

However, even for association studies, the large sample sizes necessary to study the genetics of complex disease appear unavoidable, so recent interest has focused on methods to reduce the cost. One approach is to use diallelic nucleotide bases, or single nucleotide polymorphisms (SNPs), to help identify susceptibility genes [2]. SNPs are abundantly available in the human genome (approximately 1 per kb of DNA) [3], providing a plentiful source in the genome from which to choose. Additionally, SNP genotyping can be completely automated, and recent technologies have decreased the time necessary to perform the genotyping (as reviewed by Syvanen 2001) [4]. As a result, SNPs are relatively easy, fast, and inexpensive to genotype compared to other existing technologies, such as microsatellite markers (e.g. [5,6]). A second approach to reduce the cost of genotyping is to use DNA pooling, where equal amounts of DNA from each of a group of individuals are combined and then genotyping is performed on the pool instead of on each individual's DNA separately. This procedure has the potential to substantially reduce the genotyping costs, since, if the pools are formed from *k *individuals, the genotyping costs will be reduced to (100/*k*)% of the cost of genotyping each individual.

Unfortunately, SNPs are relatively uninformative individually, i.e. more than one is required to obtain an amount of information equivalent to more informative markers, such as microsatellites. One way to increase the information from SNPs is to use haplotypes constructed from multiple SNPs, which is more powerful for detecting an association than using all SNPs individually [7]. The expectation-maximization (EM) algorithm has been implemented to obtain maximum likelihood estimates of haplotype frequencies for pooled data [8,9]. These studies show that the algorithm provides accurate estimates of the haplotype frequencies when no genotyping error is present.

More realistically, genotyping errors do occur, which can have implications for the accuracy of haplotype frequency estimates from pooled samples. In this paper we investigate the effect of genotyping error on 2-SNP haplotype frequency estimates obtained using the EM algorithm for pooled data. We show that the algorithm performs well even in the presence of genotyping error, compared to estimates obtained when there is no genotyping error present.

## Results

To evaluate the performance of the EM algorithm, the following parameters were examined: number of individuals per pool, sample size, marker allele frequency, and strength of inter-marker linkage disequilibrium. All parameters were evaluated for scenarios with and without genotyping error.

### Number of individuals (*k*) per pool

**Figure 1:**
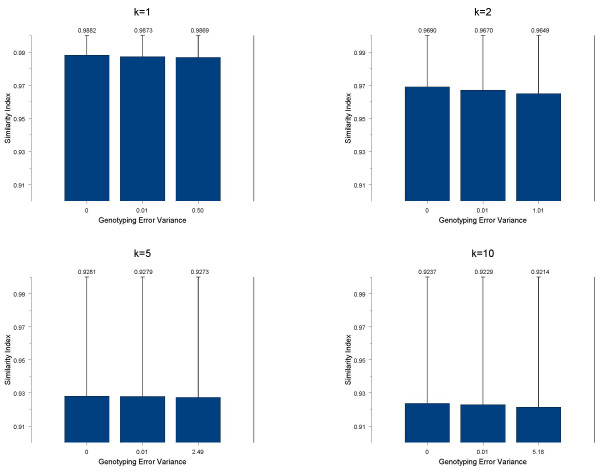
**Accuracy of the estimation procedure under different levels of genotyping error averaged over all simulated genetic models**. Accuracy of estimation procedure measured by the similarity index as a function of the level of genotyping error simulated for k = 1, 2, 5, and 10 individuals per pool.

For every scenario simulated, the accuracy of the estimates as measured by the similarity index (see below) decreased as the number of individuals per pool increased. Figure 1 shows the accuracy of the estimation procedure under different levels of genotyping error averaged over all simulated genetic models. As the genotyping error increases, the overall accuracy of the estimates slightly decreases. For pools with one individual per pool (i.e. no pooling) the error in the haplotype frequency estimates under no genotyping error is due to sampling error. Similarly, the variance of the similarity index increased with both pool size and genotyping error. That is, the variance of the similarity index for a pool of size 1 was 0.00005 and for a pool of 10 it was 0.008. Within the pool size the variance also increased with genotyping error. For example, for a pool of 10 the variance of the similarity index in the cases of no error, intermediate error, and maximum error were 0.0081, 0.0082, and 0.0084, respectively. When evaluating pool sizes greater than 1, for a given level of genotyping error the largest decrease in accuracy was between *k *= 2 and *k *= 5 individuals. There is a smaller decrease in accuracy between *k *= 5 and *k *= 10. Even though there was a larger difference in the accuracy between the pools of *k *= 2 and *k *= 5, it was minimal. Therefore, these results suggest the accuracy is approximately the same for pool sizes of 10 (92%) as it is for pool sizes of 2 (96%). That is, much less genotyping yields virtually the same amount of accuracy as would be achieved with smaller pool sizes. The figure shown is for a total sample size of 1000; the results are similar for a sample size of 500 (data not shown).

### Marker allele frequency

**Figure 2:**
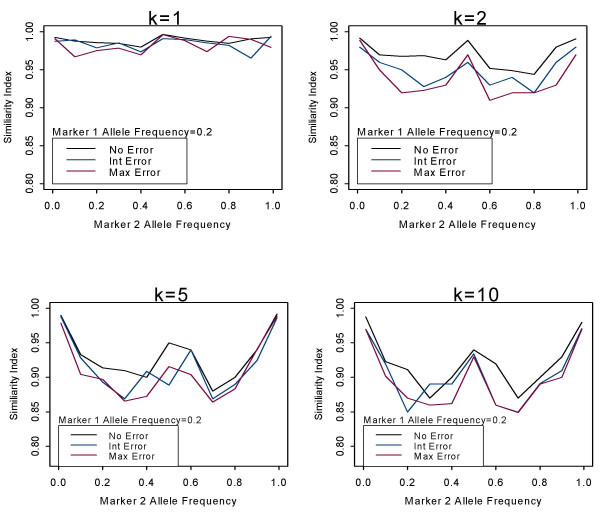
**Effect of allele frequency on accuracy of haplotype frequency estimation**. Accuracy of estimation procedure measured by the similarity index as a function of the marker 2 allele frequency for k = 1, 2, 5, and 10 individuals. The marker 2 allele frequencies range from 0.01–0.99. The black line represents no genotyping error, the blue line represents intermediate genotyping error, and the red line represents maximum level of genotyping error.

To assess the effect of allele frequency on the accuracy of the estimation procedure, haplotype frequencies were computed for simulated samples with one marker allele frequency ranging from 0.01–0.99. Figure 2 shows the plots of the similarity index as a function of marker 2 allele frequency, when marker 1 allele frequency is fixed at 0.2, for a given level of genotyping error. Overall, we see the same shape of the graphs regardless of how many individuals per pool were evaluated. That is, the allele frequencies that yield the best estimates are 0.01 and 0.99, cases when a rare allele is present. These results are for the scenario with no LD present. The graphs also peak at 0.5, but this is due to the nature of the similarity index, a measure that is automatically closer to 1 for allele frequencies close to 0.5. Therefore, as with individual data, when dealing with common allele frequencies the EM algorithm will not perform as efficiently. The figure shown is for a total sample size of 1000; the results are similar for a sample size of 500 (data not shown).

### Linkage disequilibrium

**Figure 3:**
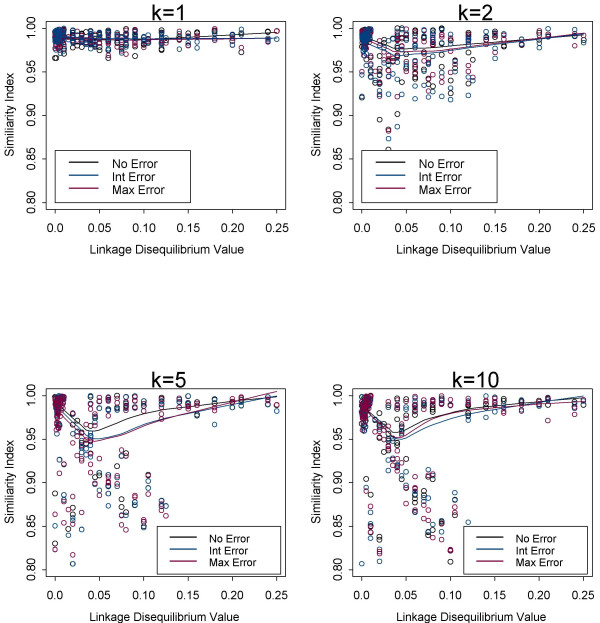
**Effect of LD on accuracy of haplotype frequency estimation for different levels of genotyping error**. Accuracy of estimation procedure measured by the similarity index as a function of the LD values for k = 1, 2, 5, and 10 individuals. LD ranges from 0.00–0.25. The black line and circles represent no genotyping error, the blue line and circles represent intermediate genotyping error, and the red line and circles represent maximum level of genotyping error. Smooth line fitted using the Lowess function in Splus.

The amount of linkage disequilibrium that is present can vary among different populations, and thus could have an effect on the accuracy of the haplotype frequency estimate. To assess the effect of LD on the haplotype frequency estimate, three levels of the LD parameter (0, δ_max_/2, and δ_max) _were investigated at different levels of genotyping error for allele frequencies ranging from 0.1–0.99 (Figure 3). Overall, the different levels of LD between the markers resulted in comparable levels of accuracy for the EM algorithm when evaluated across different pool sizes and levels of genotyping error. Figure 3 shows that the difference between the true and estimated frequencies decreases (i.e., the similarity index increases) as the amount of LD becomes stronger. Interestingly, for pooled samples the graphs in Figure 3 show that estimates achieve better accuracy if the amount of LD present is greater than 0.1 for sample sizes of 1000 individuals. We observed using sample sizes of 500 that this critical number is about 0.15 (results not shown). Therefore, as the sample sizes decreases more LD is needed to achieve the higher accuracy for pooled samples.

## Discussion

Interest continues to increase in association analysis for complex genetic traits; however, this study design is still not without shortcomings. Therefore, we evaluated the effects of genotyping error on the estimates of haplotype frequencies when pooling DNA for association studies and, more specifically, we assessed the benefits (if any) to be gained from it. Additionally, we investigated the effects of pool size, marker allele frequencies, and LD on the accuracy of the haplotype frequency estimates.

We have shown that accuracy of the haplotype frequency estimates decreases as the level of genotyping error increases. However, this decrease is small and, even in the presence of genotyping error, the estimates of the haplotype frequencies are accurate. Ideally, it would be most beneficial to design studies with a large number of individuals per pool to minimize the genotyping costs. We observed, under all genotyping error levels, that pools of size 2, 5, and 10 all achieve about the same level of accuracy. This suggests that pool sizes of 10 individuals could be used to obtain accurate estimates. Using a larger number of individuals per pool and still obtaining the same level of accuracy allows for an even greater reduction in the cost of genotyping compared to situations that utilize a smaller number of individuals per pool. Additionally, we observed that a sample size of 500 is just as accurate as a sample size of 1000 for the models simulated. Therefore this further supports the notion that less genotyping can be performed and yet the same level of accuracy obtained for haplotype frequency estimates.

We observed that marker allele frequencies and the amount of LD can have an effect on the accuracy of the haplotype frequency estimates. If a rare allele frequency is present in the pool and/or in cases of stronger LD, in the absence of genotyping error more accurate estimates are obtained. Similarly, this same pattern was observed in the presence of genotyping error. For the case of individual genotyped unrelated samples, Kirk and Cardon (2002) [10] evaluated the EM algorithm to estimate haplotype frequencies in the presence of genotyping errors using a much smaller sample size of 50. These authors observed in situations of high LD and/or when rare alleles are present that the EM algorithm offered a high degree of accuracy even in the presence of genotyping errors. In situations with low LD and/or common alleles, the EM algorithm performs more poorly for both individual and for pooled designs.

To date, the most common pooling strategy has been to create one large pool for each condition (e.g., case and control status), and to compare allele frequencies among pools (e.g. [11,12]). This strategy would certainly result in the greatest efficiency in genotyping, but at the cost that individual haplotype frequencies cannot be estimated. Under this strategy, Le Hellard et al. (2002)[13] evaluated several quantitative SNP genotyping methods for pooled samples and compared the true allele frequencies, obtained by genotyping each sample individually, to those estimated from the pooled sample. Although errors are present when estimating the allele frequencies from pooled samples, pooling provided reasonably accurate estimates, even for these very large pools. In a comprehensive review of DNA pooling, Sham (2002) [14] concludes that pooling can be considered both cost and time effective. It remains to be determined whether the cost efficiency gained by forming large pools to reduce the amount of genotyping outweighs the statistical efficiency gained by performing haplotype analysis using smaller pools.

In this analysis we chose to introduce error into genotypes because we were evaluating pooled samples. However, there are several other types of error models available for individual genotyped samples (e.g., [15,16]). Therefore, under a different error model it is possible that the conclusions reached in this analysis might have differed. Ultimately we would like to account for the genotyping error when estimating haplotype frequencies for pooled samples, just as Zou & Zhao (2003) [16] have done for individual genotyped samples.

To assess the accuracy of our results we chose the similarity index because this measure sums across all haplotypes frequencies. However, we could have chosen to evaluate the estimated haplotype frequencies individually, as in Zou & Zhao (2003) [16]. Therefore, evaluating haplotypes using a different measure could result in different conclusions. For example, in Zou & Zhao (2003) [16] the authors report four estimated haplotype frequencies to be 0.366, 0.126, 0.132, and 0.376 where the true frequencies are 0.4, 0.1, 0.1, and 0.4, respectively. Based on this, the authors note the highest change in haplotype frequency estimates to be 30% (this is from an estimated frequency of 0.132 where the true frequency is 0.1). However, for this example the similarity index, which takes all four haplotypes into account, is 0.94.

For this analysis we only chose to evaluate two-marker loci; however, our method can be extended to accommodate many marker loci. For individual samples, as the number of markers increases there is a loss of accuracy in the haplotype frequency estimates. It is possible that this loss of accuracy could be even more severe for pooled samples.

Genotyping error may have an impact on the detection of false positive or false negative signals in genetic association studies, or on the sample size needed to detect an association when using DNA pools. Gordon et al (2002) [17] quantify the effects that individual genotyping errors have on power and required sample size for case-control genetic association studies. They report that genotyping errors increase the likelihood of missing a real effect. Similarly, Zou & Zhao (2004) [18] evaluate the impact of genotyping errors on false discovery rates for individual genotyping and the impact of measurement errors or pool formation errors for pooled genotyping. They report that genotyping errors can lead to a higher rate of false positives for individual genotyping and even higher measurement errors for pooled samples.

Here we only consider the accuracy of the EM algorithm for pooled samples to estimate the haplotype frequencies in the presence of genotyping error and do not evaluate the sample size necessary to detect an association. Therefore, even though we find pool sizes as large as 10 and a sample size of 500 to be efficient for estimating haplotype frequencies, we cannot comment on the effect of genotyping errors on the ability to find false positive or negative associations.

## Conclusion

When using the EM algorithm for pooled samples, we found that genotyping error slightly decreases the accuracy of haplotype frequency estimates. However, the EM algorithm still performs well even in the presence of genotyping error. Overall, pools of 2, 5, and 10 individuals yield similar accuracy of the haplotype frequency estimates, likewise for sample sizes of 500 and 1000 individuals. Therefore, we can conclude that the overall amount of genotyping can be reduced by using 10 individuals per pool with sample sizes as small as 500 individuals.

## Methods

### Genotype simulation

Data were simulated both with and without genotyping error for each pool size (*k*) under 198 genetic models using combinations of different allele frequencies at each locus (0.01, 0.2, 0.3, 0.4, 0.5, 0.6, 0.7, 0.8, 0.9 and 0.99) and strength of LD between the markers (between 0 and 0.25). For simplicity, we only considered the case with two loci and two alleles, A_i_ and B_i_ for locus i, but this method can be extended to accommodate more marker loci. LD was measured as δ_max_, calculated as the smaller of p_A_q_B _or q_A_p_B_, where p_A _and p_B _represent allele frequencies ranging from 0.01–0.99. For each combination of allele frequencies, 3 situations were simulated with δ equal to 0, δ_max_/2, and δ_max_. Throughout our study, we assume Hardy-Weinberg equilibrium. We considered pool sizes (*k*) of 1,2,5, and 10 individuals, and total sample sizes (N) of 500 and 1000 individuals.

We used a binomial distribution as the error distribution in our simulation, with a variance (σ^2^) that depends on the parameter α. Let n_ij _be the number of times allele A at locus i is present in pool j, with possible values t_i _= 0, ..., 2k, and q_ij _be the allele frequency of allele A at marker i in pool j. Then, for each locus, the conditional probability that allele A is observed t_i _times given the true genotype was modelled as  where the summation is over values of y between 0 and 2kα where y/α rounds to t_i_. The parameter α is the genotyping error parameter. If α = 1, this represents the maximum amount of genotyping error, and as α becomes large, this distribution becomes equivalent to a having no genotyping error, as demonstrated in an example in Table 1. There are several desirable properties that this binomial distribution has, including 1) n_ij _can only take on values between 0 and 2*k*, 2) the variance of n_ij _will depend on the allele frequency, and 3) σ^2 ^can be adjusted to have a range of values.

**Table 1:** Conditional probabilities* of observed genotypes, given the true genotype of pool j. For *k *= 2 individuals with SNP locus AB in pool j.

	Observed Unordered Genotypes/Number of A Alleles in Pool j | True Unordered Genotype AABB				
α	BBBB/ 0	ABBB/ 1	AABB/ 2	AAAB/ 3	AAAA/ 4

1	0.063	0.250	0.375	0.250	0.063
5	0.0002	0.131	0.737	0.131	0.0002
25	0.000	0.006	0.988	0.006	0.000
125	0.000	0.000	1.000	0.000	0.000

* conditional probabilities computed from the binomial distribution

Based on the value of α we introduce error into genotypes by simulating three levels of genotyping error, which are defined as no genotyping error (σ^2 ^= 0), intermediate genotyping error (σ^2 ^= 0.01) which corresponds to a realistic level of genotyping error based on the results of LeHellard et al. (2002) [13], and the maximum possible genotyping error given our error distribution (σ^2 ^= 0.50–5.18, depending on the value of *k*)

### Estimation of haplotype frequencies via the EM algorithm for pooled samples

Wang et al. (2003) [8] and Ito et al. (2003) [9] independently developed algorithms to estimate haplotype frequencies utilizing an EM algorithm for pooled data. Both of these algorithms infer the estimates utilizing a maximum likelihood approach that is identical to the approach we have used when analyzing the data under no genotyping error. To investigate whether a global maximum was found, four sets of starting values were used for *k *= 1,2,5 individuals per pool, and two sets for *k *= 10, to determine if they obtain the same maximum likelihood estimate. The results we present are those from whichever starting values gave the largest maximum for the haplotype frequency estimates.

### Evaluation of haplotype frequency estimates

We compared the estimated haplotype frequencies to the true haplotype frequencies using the similarity index (I_F_) [19]. If  is the estimated haplotype frequency for haplotype i, h is the total number of haplotypes, and p_i_ is the true haplotype frequency, then I_F_ is defined as . The similarity index takes on values between 0 and 1 and is close to 0 when none of the estimated haplotype frequencies are close to the true haplotype frequencies, and 1 when all of the estimated haplotype frequencies equal the true haplotype frequencies.

## Authors' contributions

S.Q. performed the simulations, statistical analysis, and programming of the method. R.E. contributed to modeling the genotyping error. K.G. conceived the study, participated in its design and coordination, and participated in programming of the method.
